# Motion-based predictive coding is sufficient to solve the aperture problem

**DOI:** 10.1186/1471-2202-12-S1-P279

**Published:** 2011-07-18

**Authors:** Mina A Khoei, Laurent U Perrinet, Guillaume S Masson

**Affiliations:** 1 Institut de Neurosciences Cognitives de la Méditerranée, CNRS, Université de la MéditerranéeÂ , 13402 Marseille Cedex 20, France

## 

It is still unclear how information collected locally by low-level sensory neurons may give rise to a coherent global percept. This is well demonstrated in the aperture problem both in visual or haptic senses. Experimental findings on its biological solution in area MT show that local motion measures are integrated to see the dynamical emergence of global motion information [1]. We develop a theory of spatio-temporal integration defined as implementing motion-based predictive coding. This takes the form of an anisotropic, context-dependent diffusion of local information [2]. Here, we test this functional model for the aperture problem in the visual and haptic low-level sensory areas.

In our model, spatial and motion information is represented in a probabilistic framework. Information is pooled using a Markov chain formulation, merging current information and measurement likelihood thanks to a prior on motion transition. This prior is defined so that it is adapted to smooth trajectories such as are observed in natural environments.This dynamical system favors temporally coherent features. Differently to neural approximations [3], we use a particle filtering method to implement this functional model. This generalizes Kalman filtering approaches that were used previously by allowing to represent non-gaussian and multimodal distributions.

We observe the emergence of mechanisms that reflect observations made at psychophysical and behavioral levels. First, the dynamical system shows the emergence of the solution to the aperture problem and show dependence to line’s length [4]. Then,when presented with an object with a regular translation, the dynamical system grabs itsmotion independently of its shape and exhibits motion extrapolation. This shows that prediction is sufficient for the dynamical build-up of information from a local to a global scale. More generally it may give insights in the role of spatio-temporal integration on neural dynamics in the emergence of properties that are accredited to low-level sensory computations.
